# Human promyelocytic leukemia protein is targeted to distinct subnuclear domains in plant nuclei and colocalizes with nucleolar constituents in a SUMO‐dependent manner

**DOI:** 10.1002/2211-5463.12134

**Published:** 2016-10-19

**Authors:** Christian E. Lamm, Myriam Scherer, Nina Reuter, Bushra Amin, Thomas Stamminger, Uwe Sonnewald

**Affiliations:** ^1^ Division of BiochemistryDepartment of BiologyFriedrich‐Alexander University Erlangen‐NurembergGermany; ^2^ Institute for Clinical and Molecular VirologyFriedrich‐Alexander University Erlangen‐NurembergGermany; ^3^ Present address: Department of ChemistryUniversity of PittsburghPittsburghPA15260USA

**Keywords:** nucleolus, plant subnuclear structures, promyelocytic leukemia protein, nuclear bodies, small ubiquitin like modifier

## Abstract

Eukaryotic nuclei are subdivided into subnuclear structures. Among the most prominent of these structures are the nucleolus and the PML nuclear bodies (PML‐NBs). PML‐NBs are spherical multiprotein aggregates of varying size localized in the interchromosomal area. PML‐NB formation is dependent on the presence of the promyelocytic leukemia protein (PML) as well as on post‐translational modification of core components by covalent attachment of the small ubiquitin‐like modifier (SUMO). So far, PML‐NBs as well as PML have been described in mammalian cells only, whereas no orthologs are known in the plant kingdom. In order to investigate conserved mechanisms in PML targeting, we expressed human PML (hPML) fused to the GFP in *Nicotiana benthamiana*. Using confocal laser scanning microscopy and coimmunoprecipitation followed by mass spectrometric analysis, we found the fusion protein in association with nucleolar constituents. Importantly, mutants of hPML, which are no longer SUMOylated, showed altered localizations, implying SUMO‐dependent targeting of hPML in plants as has previously been shown for mammalian cells. Interestingly, in the presence of proteasome inhibitors, hPML could also be found in the nucleolus of mammalian cells suggesting conserved targeting mechanisms of PML across kingdoms. Finally, *Solanum tuberosum* COP1, a proposed PML‐like protein from plants, was fused to the red fluorescent protein (RFP) and coexpressed with hPML::eGFP. Microscopic analysis confirmed the localization of COP1::RFP in nuclear speckles. However, hPML::eGFP did not colocalize with COP1::RFP. Hence, we conclude that plants do not possess specialized PML‐NBs, but that their functions may be covered by other subnuclear structures like the nucleolus.

**Database** Proteomics data have been deposited to the ProteomeXchange Consortium with the identifier PXD004254.

**Abbreviations**
COP1: constitutive photomorphogenic protein 1
FIB2: fibrillarin 2
hPML: human promyelocytic leukemia protein
PML‐NB: PML nuclear bodies
SUMO: small ubiquitin‐like modifier

Although animal and plant nuclei are superficially similar, they differ in their compositional details. While it has long been known that eukaryotic nuclei in general contain further subnuclear structures like nucleoli or cajal bodies which are well known in both kingdoms, other structures seem to be less conserved 1, 2. One prominent example for a subnuclear domain characterized in metazoans but not in plants are structures termed PML nuclear bodies (PML‐NBs). These dot‐like structures of varying size, which are also known as nuclear domain 10 (ND10) or PML oncogenic domains (POD), are localized to the interchromosomal area and are characterized by a subset of interacting core proteins 3. Among these is the eponymous tumor‐suppressor protein, promyelocytic leukemia protein (PML), as well as Sp100 and Daxx 1, 4, 5. Importantly, the integrity of PML‐NBs is dependent on the presence of PML as well as its post‐translational modification by small ubiquitin‐like modifier (SUMO) proteins. Also, the ability of PML and other PML‐NB constituents to bind SUMO noncovalently is essential for the efficient formation of PML‐NBs 5, 6, 7, 8. The suggested functions conferred by this subnuclear multiprotein structure are manifold: Due to the large number of in part transiently associated cellular proteins, PML nuclear bodies were considered as sites of nuclear storage or as a dump for excess proteins 9. The fact that PML is reacting toward the application of heat stress with redistribution into smaller aggregates, led to the belief that PML‐NBs are also involved in a general heat stress response 10. However, in recent years these subnuclear structures were successively assigned to several other, more active, functions. Since PML knockout mice exhibit lower genomic stability and an increased liability to tumor formation during exposure to carcinogens, a role in DNA damage repair as well as tumor suppression was proposed 11, 12. Interestingly, PML‐NBs were also implicated in antiviral defense and thus also pose a target for viral effectors 9, 13, 14, 15.

To date, no clear plant ortholog of PML has been found and it remains uncertain whether PML‐NBs exist in plants. However, work in *Arabidopsis thaliana* identified the constitutive photomorphogenic protein 1 (COP1) as a potential functional homolog of PML 16. As the name suggests, the plant protein is an important negative regulator in plant photomorphogenesis. When grown in darkness, wild‐type seedlings undergo etiolation 17, while cop1‐knockout mutants do not undergo etiolation, but show a *constitutive photomorphogenic* developmental pattern 18, 19. The postulated functional analogy of COP1 and PML is based on similar localization patterns and domain architecture, since both *A. thaliana* COP1 (AtCOP1) and human PML (hPML) localize to the nucleus in a speckled manner and both contain an N‐terminal RING‐finger with an adjacent coiled‐coil domain 16. However, the primary sequences of COP1 and hPML do not show significant similarity, and COP1 is missing B‐Box domains present in hPML, while hPML does not possess WD40 repeats found at the C terminus of COP1 20. Furthermore, Wang *et al*. 21 could identify a COP1 homolog in mammalian cells which could be shown to interact with Jun transcription factors. Interestingly, the mammalian COP1 homolog exhibited a light‐dependent shift in localization, similar to the *Arabidopsis* protein 22, 23. Based on the described inconsistencies, a functional commonality of hPML and AtCOP1 seemed unlikely and therefore it has been suggested that PML is not evolutionarily conserved 20.

To address this unresolved question, we investigated the targeting and SUMOylation status of hPML in plant cells. In *Nicotiana benthamiana*, the protein could be shown to localize to interchromosomal areas and to colocalize to some extent with the nucleolus/cajal body‐marker fibrillarin 2 (FIB2) when heterologously expressed 24. Of note, a nucleolar localization could also be found in mammalian cells upon stress treatment. Moreover, coimmunoprecipitation of hPML::eGFP allowed us to identify 29 associated plant nuclear or nucleolar proteins. Strikingly, some of these proteins are also known to reside in the hPML‐interactome in human cells. Furthermore, hPML mutants exhibiting impaired SUMOylation 8 showed mistargeting of the protein, suggesting that SUMOylation is important for the nucleolar localization of hPML in plant cells. Finally, colocalization experiments with hPML and plant COP1 revealed both proteins to reside in distinct subnuclear domains, supporting the previous evaluation of COP1 to be not functionally redundant to hPML and suggesting that PML‐NBs may not be conserved across kingdoms.

## Results

### Human PML is targeted to interchromosomal subnuclear domains and interacts with nuclear and nucleolar proteins *in planta*

**Figure 1:**
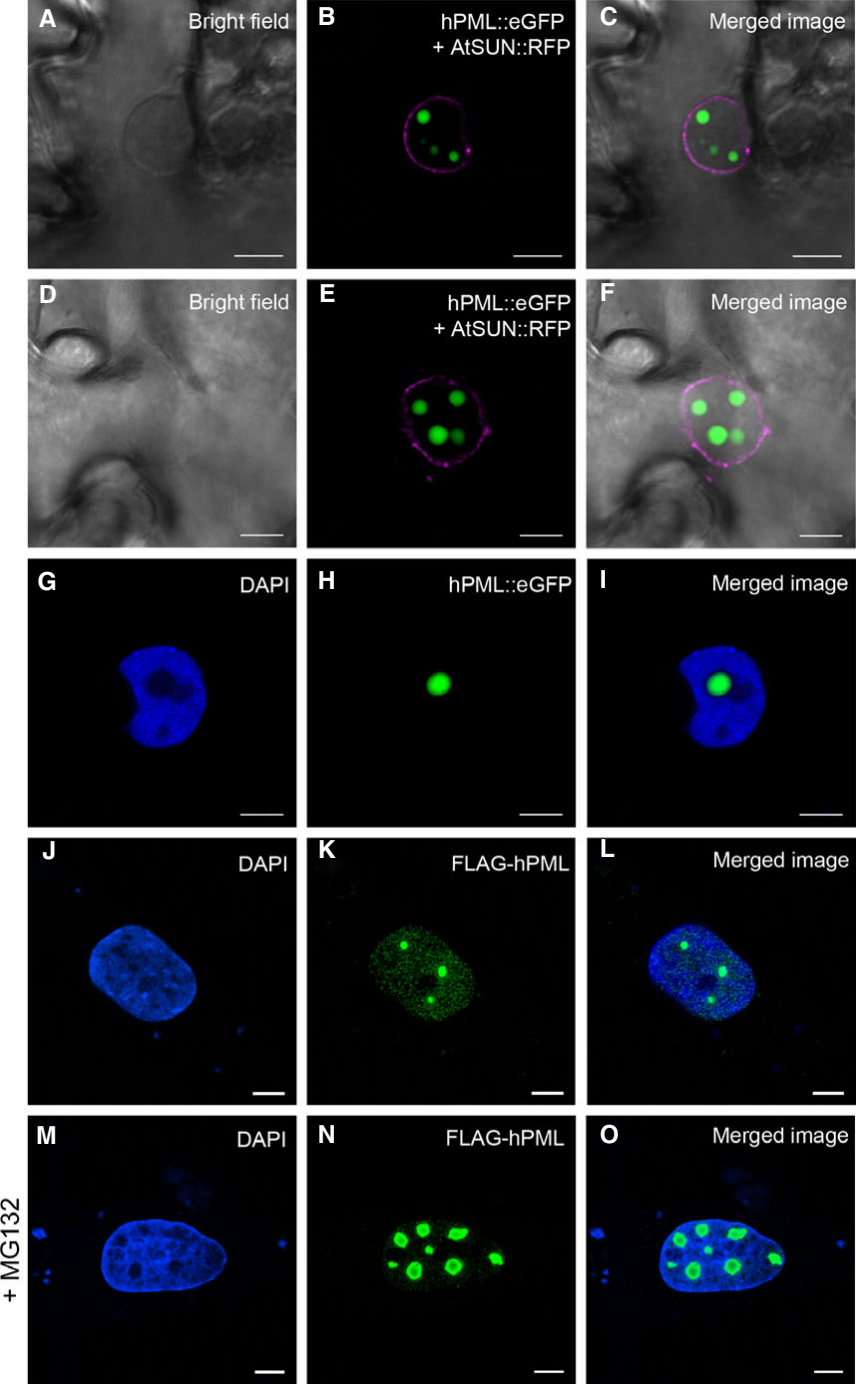
Subnuclear localization of hPML. (A–F) Expression of hPML::GFP and AtSUN::RFP in *Nicotiana benthamiana*. (A, D) Bright field images. (B), (E) hPML::eGFP accumulated in spherical, subnuclear structures (green); AtSUN::RFP was used to stain the inner nuclear membrane (magenta). (C, F) Merged images from (A) and (B) or (D) and (E), respectively. (G–I) 4′,6‐diamidino‐2‐phenylindole (DAPI) costaining indicated an interchromosomal localization of hPML::eGFP in *N. benthamiana*. Images show a single optical slice derived from a Z‐stack. (G) DAPI staining of the plant nucleus revealed chromatin‐rich areas. (H) Subnuclear aggregate of hPML::eGFP. (I) The fluorescence signal derived from the hPML‐construct localized to areas devoid of DAPI‐staining. (J–O) Expression of FLAG‐tagged hPML isoform VI in primary human fibroblasts with a siRNA‐mediated depletion of endogenous PML protein in the absence (J–L) or presence (M–O) of the proteasome inhibitor MG132. (J, M) DAPI staining of cell nuclei revealing the subnuclear localization of nucleoli; (K, N) Indirect immunofluorescence staining using an anti‐FLAG antibody to detect FLAG‐tagged PMLVI; (L, O) Merged images of (J) and (K) or (M) and (N), respectively. White bars represent 5 μm.

**Figure 2:**
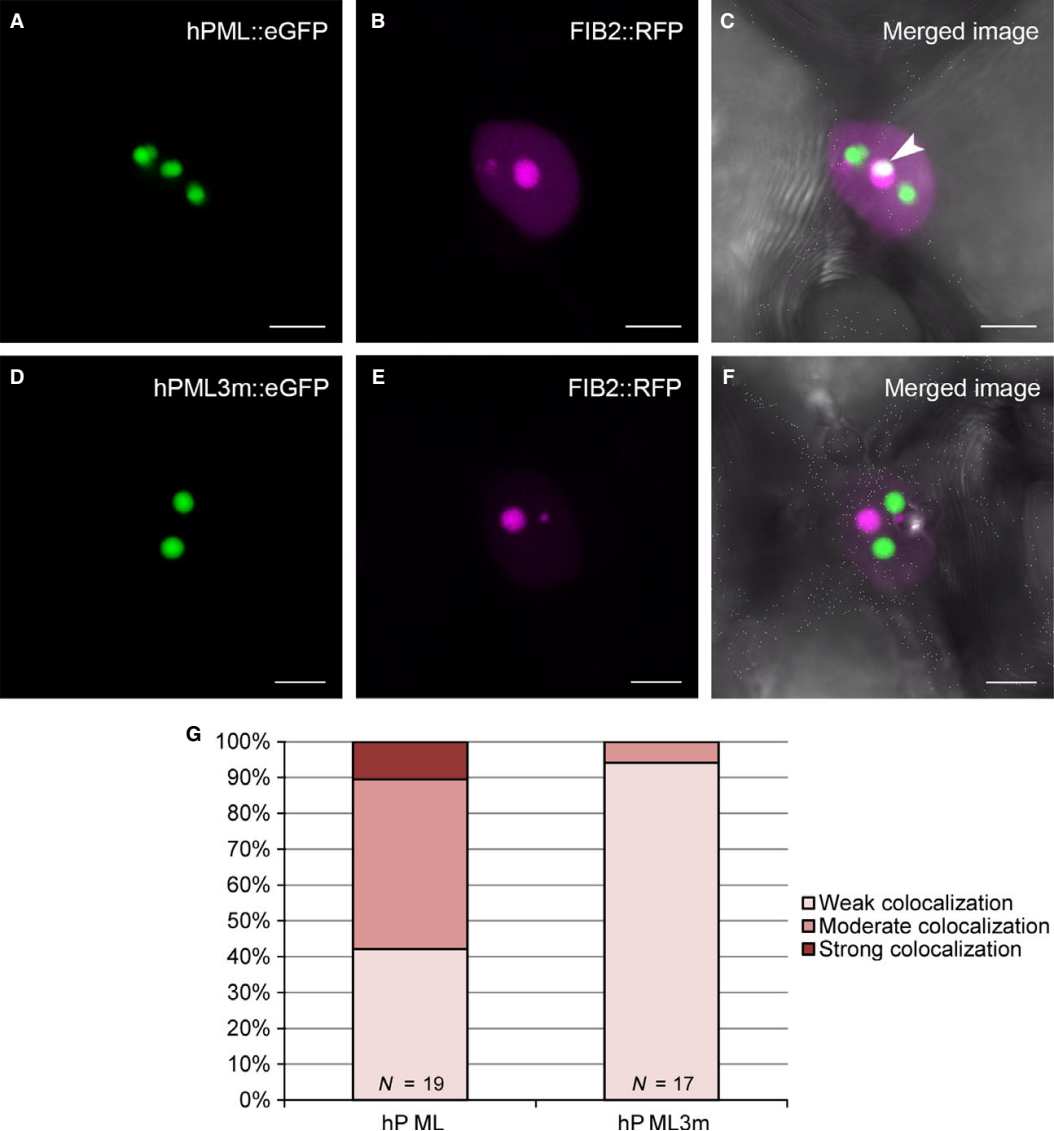
Colocalization of hPML::eGFP, hPML3 m::eGFP, and FIB2::RFP. (A–C) Coexpression of hPML::eGFP and FIB2::RFP in *Nicotiana benthamiana*. Images show a maximum projection of a Z‐stack image series. (A) hPML::eGFP exhibits the described subnuclear localization pattern. (B) FIB2::RFP accumulates in the nucleolus and in the cajal bodies. (C) Merged image of (A) and (B) reveals an area of extensive colocalization of hPML::eGFP and FIB2::RFP indicated by an arrowhead. (D–F) Coexpression of hPML3 m::eGFP and FIB2::RFP in *N. benthamiana*. The representative images show a maximum projection of a Z‐stack image series. (D) hPML3 m::eGFP still forms spherical subnuclear structures. (E) FIB2::RFP staining of nucleolus and cajal bodies. (F) Merged image shows no obvious colocalization of hPML3 m::eGFP and FIB2::RFP. (G) Statistical colocalization analysis based on Pearson's correlation coefficient and classification according to Zinchuk *et al*. 29 of 19 or 17 nuclei expressing hPML::eGFP and FIB2::RFP or hPML3 m::eGFP and FIB2::RFP, respectively. Colocalization potential of hPML and FIB2 is abolished in the hPML3 m mutant in plant cells, indicating a role of protein SUMOylation in subnuclear targeting.

**Figure 3:**
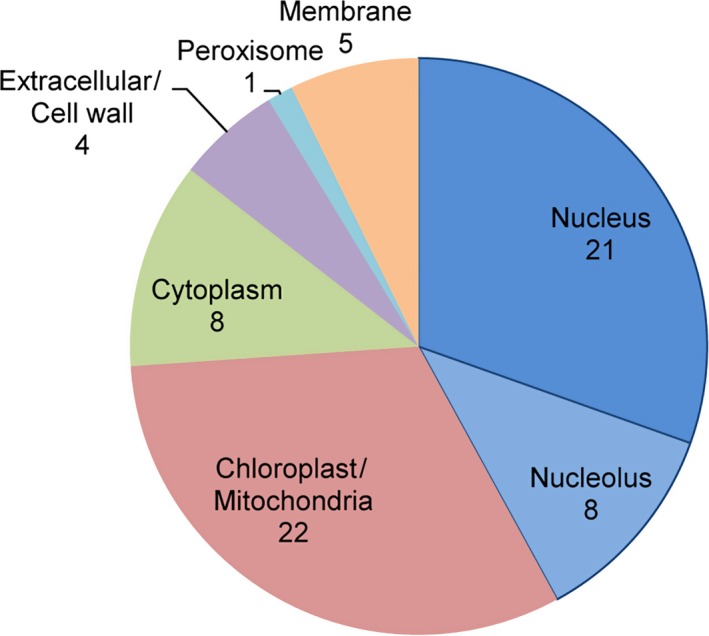
Subcellular localization of plant proteins identified via PML immunoprecipitation. The respective *Arabidopsis thaliana* homolog of each *Nicotiana benthamiana* protein was determined by BLAST analysis, the localization was adopted from http://www.arabidopsis.org. Numbers indicate the quantity of identified proteins localized to the respective organelle.

In order to address the still unsettled question whether PML‐NBs are present in plant nuclei, we fused the coding region of hPML isoform VI with eGFP to generate the chimeric construct hPML::eGFP 25. Using correspondingly transformed *Agrobacterium tumefaciens*, the protein was expressed in *N. benthamiana* leaves together with the nuclear envelope marker AtSUN1::RFP 26. By means of confocal laser scanning microscopy, we found the fusion protein to form spherical structures of varying size in the nucleus (Fig. 1A–F). However, the observed structures did not resemble PML‐NBs as present after expression of FLAG‐tagged hPML isoform VI in mammalian cells (Compare Fig. 1J–L), since they were larger and appeared in fewer numbers. Staining of leaves expressing hPML::eGFP with 4′,6‐diamidino‐2‐phenylindole (DAPI) revealed the human protein to be targeted to interchromosomal areas (Fig. 1G–I), and colocalization studies with FIB2::RFP 27 indicated partial nucleolar association (Fig. 2A–C). A localization pattern similar to the typical dot‐like pattern of PML‐NBs in mammalian cells could not be observed. However, upon treatment of mammalian cells with the proteasome inhibitor MG132, hPML isoform VI was found to relocalize to the nucleolus, a finding which has previously been described for endogenous hPML in a variety of mammalian cell lines (Fig. 1M–O) 28. To further ascertain this result and to reveal hPML‐associated plant proteins, we conducted a coimmunoprecipitation of hPML::eGFP followed by mass spectrometric analysis. In total, 69 proteins were identified being significantly (> 13× ‐10lg P) and more than five times enriched in samples containing hPML::eGFP (Table S1). Of these 69 proteins, 22 were annotated as chloroplastic or mitochondrial. Eight of the identified proteins were assigned to the cytoplasm, five were membrane proteins, four were annotated as extracellular or cell wall‐associated, and one was annotated as peroxisomal. The remaining 29 proteins were annotated as nuclear proteins, of which 8 could be assigned to the nucleolus (Fig. 3). Foremost, the high number of nucleolar and nuclear proteins underlines the result obtained in the microscopical investigation and confirms hPML::eGFP association with constituents of the plant nucleolus. Strikingly, several of the identified nuclear proteins (or proteins related to them) have previously been described to interact with hPML in mammalian cells (Table 1). This finding suggests that some interactions are conserved in mammalian and plant cells and point to general functions.

**Table 1:** Nuclear *Nicotiana benthamiana* proteins identified as potential PML interactors via immunoprecipitation. Description and Accession number adopted from the *N. benthamiana* protein database, Best BLAST hit in *Arabidopsis* shows the respective locus identifier, Associated human PML interactor indicates proteins that interact with PML in mammalian cells as stated in the respective References

Description	Accession Number *N. benthamiana* database	Best BLAST hit *Arabidopsis*	Annotated localization	Associated human PML interactor	Reference
Nuclear pore complex protein NUP85	Niben101Scf01611g04026.1	AT4G32910	Nuclear Pore Complex		
ATP‐dependent RNA helicase	Niben101Scf06884 g00001.1	AT5G62190	Nucleolus		
Holliday junction ATP‐dependent DNA helicase RuvB	Niben101Scf01281 g02006.1	AT5G67630	Nucleolus		
Importin subunit alpha	Niben101Scf03390 g08002.1	AT3G06720	Nucleolus		
Nucleolar protein 16	Niben101Scf04623 g02007.1	AT1G02870	Nucleolus		
Replication factor C subunit 3	Niben101Scf01888 g07004.1	AT1G21690	Nucleolus	Replication factor C4	McNamara *et al*. 33
Replication factor C subunit 3	Niben101Scf02197 g01014.1	AT1G77470	Nucleolus	Replication factor C4	McNamara *et al*. 33
Ribosomal RNA large subunit methyltransferase	Niben101Scf23821 g00012.1	AT3G28460	Nucleolus		
RNA‐binding protein like	Niben101Scf15360 g00006.1	AT4G32720	Nucleolus		
2‐oxoglutarate (2OG) and Fe(II)‐dependent oxygenase superfamily protein	Niben101Scf06267 g03010.1	AT2G17970	Nucleus		
65‐kDa microtubule‐associated protein 6	Niben101Scf07839 g00002.1	AT2G01910	Nucleus	Microtubule‐associated protein 1 light chain 3 beta	He *et al*. 40
ATP‐dependent RNA helicase	Niben101Scf05387 g09003.1	AT3G06980	Nucleus		
DNA‐directed RNA polymerase subunit D	Niben101Scf00414 g09001.1	AT1G60620	Nucleus	DNA‐directed RNA polymerase POLR2E	Ravasi *et al*. 35
FRIGIDA‐like protein 3	Niben101Scf04181 g00024.1	AT5G48385	Nucleus		
Heavy metal transport/detoxification superfamily protein	Niben101Scf07109 g05004.1	AT4G35060	Nucleus		
Histone‐lysine *N*‐methyltransferase	Niben101Scf01020 g00014.1	AT5G04940	Nucleus	Histone‐lysine *N*‐methyltransferase G9a	Lusic *et al*. 34
Histone‐lysine *N*‐methyltransferase 2A	Niben101Scf12382 g00017.1	AT1G73100	Nucleus	Histone‐lysine *N*‐methyltransferase G9a	Lusic *et al*., 34
Holliday junction ATP‐dependent DNA helicase RuvB	Niben101Scf04267 g00016.1	AT5G27740	Nucleus	Replication factor C4	McNamara *et al*. 33
Nuclear cap‐binding protein subunit 1	Niben101Scf07629 g00001.1	AT2G13540	Nucleus		
Plastid transcriptionally active 12	Niben101Scf08698 g02020.1	AT2G34640	Nucleus		
Protein IQ‐DOMAIN 31	Niben101Scf06172 g01003.1	AT1G74690	Nucleus		
Protein IQ‐DOMAIN 31	Niben101Scf01326 g08021.1	AT1G74690	Nucleus		
Replication factor C subunit 3	Niben101Scf07391 g04027.1	AT1G63160	Nucleus	Replication factor C4	McNamara *et al*. 33
Replication factor C subunit 4	Niben101Scf01382 g04008.1	AT5G22010	Nucleus	Replication factor C4	McNamara *et al*. 33
Replication factor C subunit 5	Niben101Scf05056 g02015.1	AT1G63160	Nucleus	Replication factor C4	McNamara *et al*. 33
RNA‐binding KH domain‐containing protein	Niben101Scf03015 g05008.1	AT5G15270	Nucleus		
RNA‐binding protein 1	Niben101Scf06105 g03006.1	AT4G10110	Nucleus		
snRK1‐interacting protein 1	Niben101Scf03949 g06008.1	AT1G71310	Nucleus	RAD51	Boichuk *et al*. 38
WD‐40 repeat family protein/small nuclear ribonucleoprotein	Niben101Scf11084 g02025.1	AT2G41500	Nucleus		

### SUMOylation‐negative hPML mutants show aberrant localization patterns

**Figure 4:**
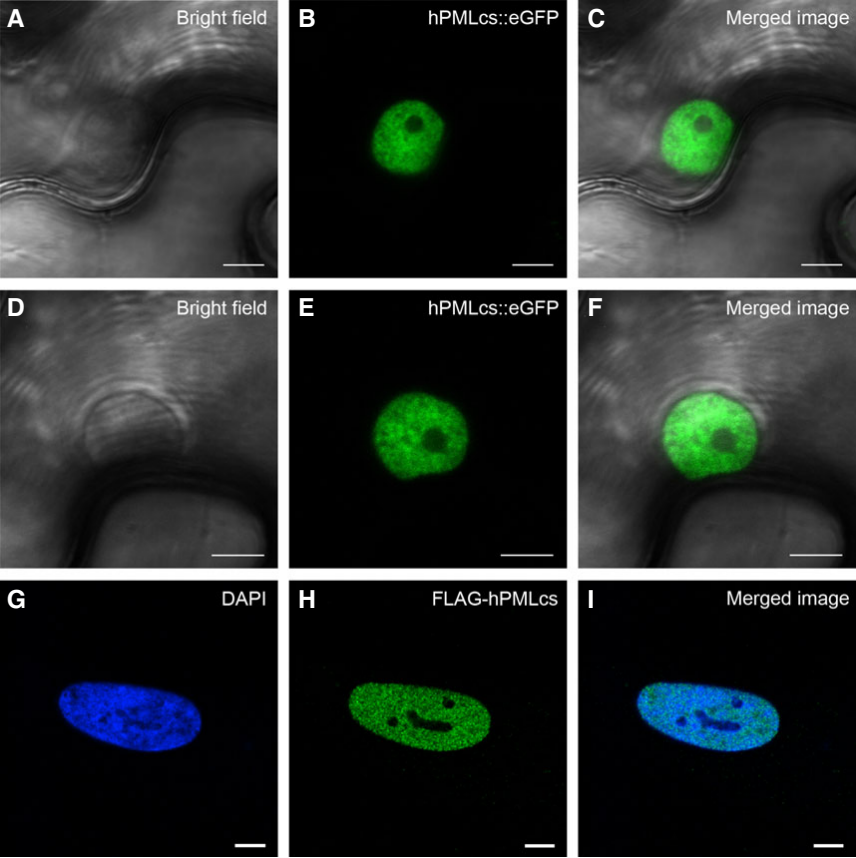
Localization of the SUMOylation‐deficient hPMLcs‐mutant. (A–F) Expression of hPMLcs::eGFP in *Nicotiana benthamiana*. (A, D) Bright field images. (B, E) The hPMLcs::GFP fusion construct is dispersed throughout the nucleoplasm, but is not present in the nucleolus. (C, F) Merged images from (A) and (B) or (D) and (E), respectively. White bars represent 5 μm. (G–H) Expression of FLAG‐tagged hPMLcs in primary human fibroblasts with a siRNA‐mediated depletion of endogenous PML protein. (G) 4′,6‐diamidino‐2‐phenylindole staining of the cell nucleus; (H) Indirect immunofluorescence staining of FLAG‐tagged hPMLcs using an anti‐FLAG antibody; (I) Merged image of (G) and (H). White bars represent 5 μm.

In mammalian cells, proper targeting of hPML is dependent on SUMOylation of the protein. To test whether targeting of hPML::eGFP in plant cells may also be SUMO‐dependent, we generated eGFP fusion constructs of hPML mutants termed hPMLcs (containing two cysteine to serine amino acid exchanges in the protein's RING domain) and hPML3 m (in which the three known lysine residues modified by SUMO are changed to arginines). Both mutants have previously been shown to be SUMOylation‐negative and not to be able to induce formation of proper PML‐NBs 8. In mammalian cells, FLAG‐tagged hPMLcs show a dispersed localization throughout the nucleoplasm (Fig. 4G–I). Interestingly, upon expression of hPMLcs::eGFP in *N. benthamiana*, we observed a localization pattern comparable to the situation observed in mammalian cells (Fig. 4A–F). The hPMLcs::eGFP fusion protein was found in the nucleoplasm in a granular pattern, but not in the nucleolus. Expressed in mammalian cells, the SUMOylation mutant, hPML3 m, is still able to form subnuclear aggregates, but not functional PML‐NBs 8. In plant cells, hPML3 m::eGFP could also be localized in subnuclear aggregates similar to the wild‐type protein. However, coexpression of hPML3 m::eGFP and FIB2::RFP revealed that the protein does not colocalize with the nucleolar marker, FIB2 (Fig. 2D–F). This could also be confirmed by statistical validation of the colocalization of hPML::eGFP, hPML3 m::eGFP, and FIB2::RFP. To this end, Z‐stack images of coexpressing cells were evaluated according to the classification by Zinchuk *et al*. 29. Here, approximately 58% of all screened nuclei showed strong or moderate colocalization of hPML::eGFP and FIB2::RFP, whereas hPML3 m::eGFP colocalized only in 5.9% of all observed nuclei to a moderate extent with the nucleolus marker, while the remainder exhibited only weak colocalization (Fig. 2G). Taken together, this indicates that the hPML RING domain represents an important factor for the subnuclear localization of the protein, not only in mammalian cells but also in *N. benthamiana*. Furthermore, the reduced ability of hPML3 m::eGFP to associate with FIB2::RFP suggests that SUMOylation of hPML is an important determinant for nucleolar targeting in plants and suggests an evolutionarily conserved function of SUMOylation in targeting of proteins to subnuclear domains.

### hPML::eGFP is SUMOylated, while hPMLcs::eGFP and hPML3 m::eGFP exhibit impaired SUMOylation *in planta*

**Figure 5:**
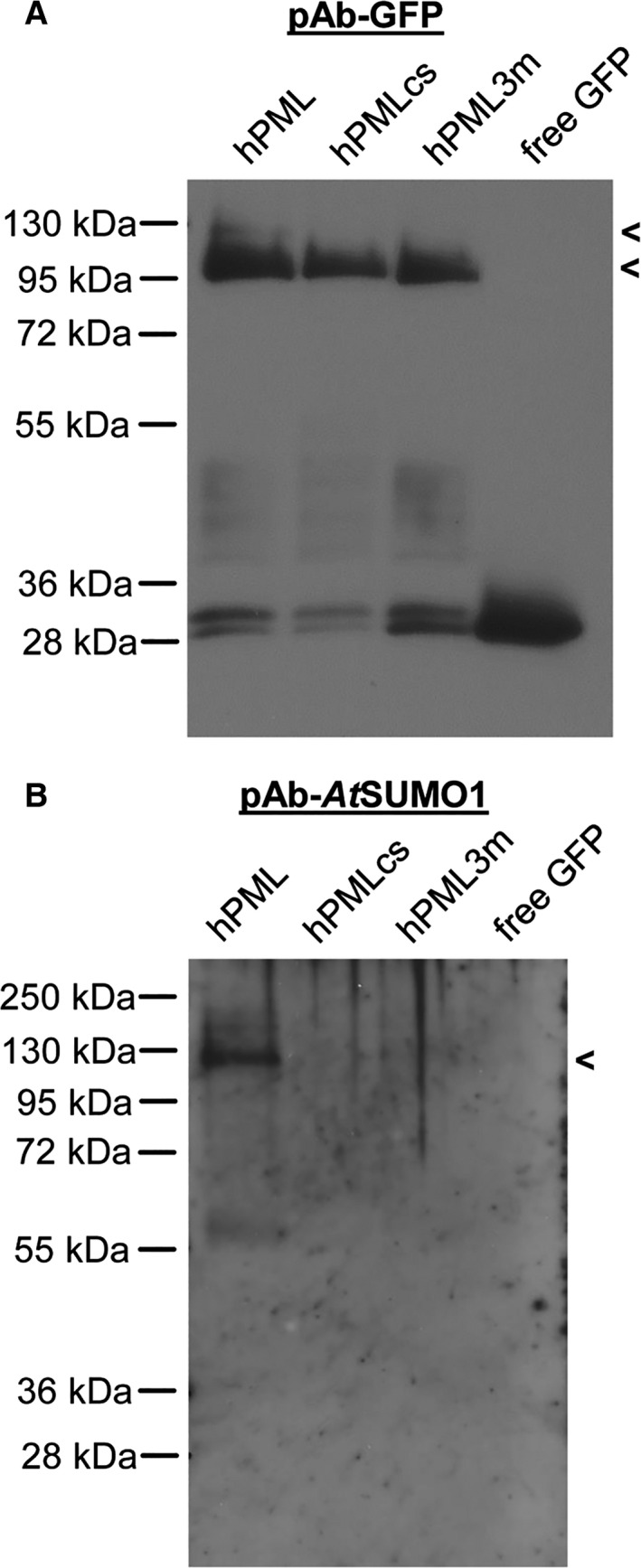
Western blot analysis of immunoprecipitated hPML constructs. (A) *Nicotiana benthamiana* leaves expressing hPML::GFP, hPMLcs::GFP, hPML3 m::eGFP, and free GFP were subjected to immunoprecipitation followed by western blotting. Successful pulldown of target proteins was confirmed by immunodetection using a polyclonal anti‐GFP antibody. hPML::eGFP as well as hPMLcs::eGFP and hPML3 m::eGFP were efficiently precipitated with a specific band migrating at approximately 100 kDa. An additional faint band could be observed at ca. 130 kDa for hPML::eGFP (Indicated by brackets). The free GFP control yielded one band at ca. 30 kDa. (B) Aliquots of the same samples used in (A) were equally loaded on an individual gel and subjected to western blotting followed by immunodetection using an anti‐*At* SUMO‐1 antibody. For hPML::eGFP, a single band could be detected migrating at ca. 130 kDa, identifying the band observed in (A) as SUMOylated form of hPML. In contrast to this, no SUMOylation of the hPML mutants could be detected.

To verify SUMOylation of hPML *in planta*, protein extracts of *N. benthamiana* leaves expressing either hPML::eGFP, hPMLcs::eGFP, hPML3 m::eGFP, or free GFP were subjected to immunoprecipitation followed by western blot analysis. Target protein precipitation and correct expression of the chimeric gene constructs was determined by using a polyclonal anti‐GFP antibody. As indicated in Fig. 5A, hPML::eGFP, hPMLcs::eGFP, and hPML3 m::eGFP proteins were efficiently precipitated and accumulated to comparable amounts at approximately 100 kDa. Interestingly, hPML::eGFP showed a faint additional band at ca. 130 kDa, which was absent in hPMLcs::eGFP and hPML3 m::eGFP. Subsequently, potential SUMOylation of the precipitated proteins was tested with an individual blot using a polyclonal antibody directed against *A. thaliana* SUMO‐1. For hPML::eGFP, a distinct band could be detected at approximately 130 kDa (Fig. 5B), thus running at the same height as the faint band observed before. Hence, this higher molecular band most likely represents SUMOylated hPML::eGFP. In contrast to this, hPMLcs::eGFP as well as hPML3 m::eGFP did not yield bands migrating at this height. In good accordance with the study initially characterizing the mutant 8, this indicates an abolished SUMOylation state of both hPML mutants in *N. benthamiana*.

### Human PML and plant COP1 are targeted to individual subnuclear structures

**Figure 6:**
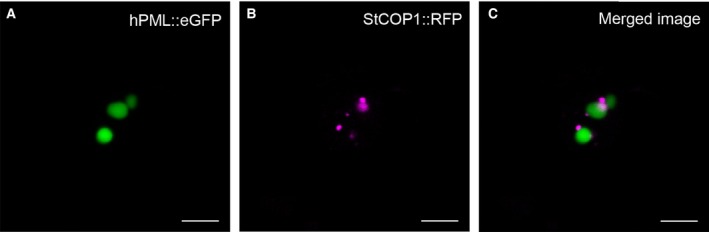
Coexpression of hPML::eGFP and StCOP1::RFP in *Nicotiana benthamiana*. (A) hPML::eGFP localized to spherical structures as observed before. (B) StCOP1::RFP was found in nuclear speckles, as was shown in previous studies. (C) Merged image of (A) and (B) revealed that both proteins were not present in the same subnuclear domain, arguing for diverging functions. However, a close spatial relation could be seen, hinting toward a possible dynamic exchange of contents.

Since the suggested functional analogy of plant COP1 to hPML is still under debate, 16, 20, we wanted to investigate whether both proteins would colocalize in the same subnuclear compartment in plant cells. Therefore, we coexpressed hPML::eGFP and *Solanum tuberosum* COP1::RFP in *N. benthamiana* leaves. As described above, hPML::eGFP showed a nucleolar localization, while COP1::RFP was present in independent subnuclear domains (Fig. 6). Although both signals did not overlap, both domains were intriguingly adjoining. Therefore, a relation of both proteins to some extent cannot be excluded. Their spatial proximity might even hint at a dynamic exchange of contents between both subnuclear structures, which, considering the dynamic nature of nuclear bodies, might not be surprising 2. However, the largely missing colocalization of hPML::eGFP and COP1::RFP is further evidence for diverging roles of the respective structures in the nucleus.

## Discussion

In mammalian cells, the subnuclear structures known as PML‐NBs are intensively studied and implicated in various cellular processes. In the plant kingdom, however, these structures are largely uncharacterized. In this study, we utilized hPML for heterologous protein expression experiments in order to test for the possible presence of PML‐NBs in *N. benthamiana* cells.

Upon expression of an hPML::eGFP construct, we could show targeting of the human protein to subnuclear interchromosomal regions associated with the nucleolus, but not to punctuate structures comparable to PML‐NBs in the mammalian system 3. Of note, association of hPML with the nucleolus could be shown to different degree in human cell lines: In senescent human mesenchymal stem cells and human skin fibroblasts, but not in immortalized cell lines, hPML may be found in patterns adjacent to nucleoli or nucleolar elements differing from regular PML‐NBs 30. Similarly, various forms of cellular stress like UV‐ or γ‐radiation as well as chemical inhibition of topoisomerase II, RNA polymerase I and the proteasome lead to a redistribution of hPML in favor of an association with nucleolar structures or constituents. Interestingly, a nucleolar targeting domain was identified at the C‐terminal part of hPML isoform I, which was suggested to be auto‐inhibited by the adjacent SUMO‐interacting motif 28, 31. Although the hPML isoform VI used in this study does not contain this C‐terminal part responsible for hPML isoform I nucleolar targeting, it still relocalized to the nucleolus upon treatment of mammalian cells with proteasome inhibitors 25. This may indicate that additional protein interactions contribute to the nucleolar targeting of hPML. Nevertheless, our observations underline the dynamic nature of subnuclear structures and connect hPML functions with the nucleolus, indicating that the observed localization of hPML::eGFP in *N. benthamiana* is reasonable.

Coimmunoprecipitation of proteins associated with hPML::eGFP revealed a large number of nuclear and nucleolar proteins supporting the microscopical analysis. Among those, several proteins known from the hPML‐interaction network in human cells could be identified. This indicates conserved protein–protein interactions between mammalian and plant cells. In this regard, the list of nuclear and nucleolar proteins contained five proteins annotated as replication factor C subunits and one protein (annotated as Holliday junction ATP‐dependent DNA helicase RuvB) that shows high sequence similarity to *Arabidopsis* replication factor C 3 (At5G27740). Two of the former five proteins showed the highest similarity (based on BLAST analyses) to the identical *Arabidopsis* protein (Gene locus identifier: At1G63160). Interestingly, an interaction of human replication factor C4 with the hPML/RARα fusion protein relevant in acute promyelocytic leukemia could be shown before 32. Other plant nuclear proteins found in the immunoprecipitation include a DNA‐directed RNA polymerase subunit as well as two histone‐lysine *N*‐methyltransferases. Of note, the interaction with the human DNA‐directed RNA polymerase POLR2E was demonstrated by two‐hybrid experiments, while the interaction of hPML with the histone‐lysine *N*‐methyltransferase G9a could be shown via immunoprecipitation in HEK293 cells 33, 34. Notably, the *Arabidopsis* histone‐lysine *N*‐methyltransferase SDG8 could be identified in a screening for plant SUMO‐interacting proteins 35. The coimmunoprecipitation also yielded a protein annotated as snRK1‐interacting protein 1, which shows high similarity to *Arabidopsis* RADIATION‐SENSITIVE 52‐1 (RAD52), a protein involved in DNA double‐strand break repair 36. Strikingly, an involvement of hPML in DNA double‐strand repair as well as an interaction of hPML isoforms V and I with the RAD52‐interactor RAD51 was shown in human cell lines 37, 38. Another interesting finding is the 65‐kDa microtubule associated protein 6, since interaction of hPML with a human microtubule‐interacting protein (microtubule‐associated protein 1 light chain 3 beta) was suggested to be important for cell growth control 39. Some of the proteins identified in the present experiment may also give hints toward the intracellular transport of hPML, like the importin subunit alpha or the nuclear pore complex protein NUP85, whereas some other candidates on the list remain enigmatic and need further investigation. Considering the absence of structures resembling human PML‐NBs in plant cells expressing hPML::eGFP but keeping the high number of coimmunoprecipitated nucleolar proteins in mind, it is tempting to speculate about the nucleolus as a site of hPML‐associated functions in plants. This assumption is further fortified by the loss of nucleolar association of hPML in *N. benthamiana*, when its SUMOylation is impaired by mutating the target lysine residues 8, 25. Moreover, the dependency of the protein's SUMOylation status for its subnuclear localization could be one starting point to investigate a SUMO‐mediated nucleolar‐targeting mechanism in plants, a mechanism which is already known from human topoisomerase I as well as Werners helicase. Here, SUMOylation leads to redistribution from the nucleolus to the nucleoplasm 40, 41, 42. In addition, large amounts of SUMO‐1 are found at the nuclear envelope and in the nucleolus of mammalian cells 43. Furthermore, the SUMO‐deconjugating sentrin/SUMO‐specific protease SENP5 as well as its close homolog SENP3 are concentrated in the nucleolus, where they are involved in cell division and rRNA processing, respectively 44, 45, 46, 47. Also, a proteomic study revealed a set of nucleolar SUMO target proteins, reflecting the tight interconnection of this post‐translational modification and the nucleolus 48. In plants, the important role of SUMO is also becoming apparent. SUMOylation has been implicated in processes ranging from stress responses, pathogen defense, and abscisic acid signaling to flower induction 49, 50, 51, 52, 53, 54. Importantly, the involvement of the plant nucleolus in SUMO‐related processes is underlined by the identification of several SUMO‐interacting proteins located in the nucleolus 35. In regard of the drastic shift of hPML localization both in plants as well as in mammalian cells when mutating two cysteine residues in the RING domain, it is important to note that the RING domain was shown to be a mediator of protein–protein interactions, among others with the E2 SUMO‐1‐conjugating enzyme, UBC9 55. Taking into account that hPMLcs::eGFP was not SUMOylated anymore *in planta*, it seems reasonable that the RING domain is mediating post‐translational modification of hPML with SUMO. Whether the observed mistargeting of hPMLcs::eGFP is an effect of impeded protein–protein interaction or a consequence of effective blocking of SUMOylation remains unclear.

Taken together, our data indicate that human PML is targeted to subnuclear domains in the plant nucleus, where it associates with nucleolar constituents dependent on the integrity of its RING domain and its SUMOylation status. It is able to interact with several factors also present in the hPML‐interactome in human cells. Since we were not able to identify distinct hPML‐containing subnuclear structures resembling PML‐NBs in plants, we suggest that some PML‐NB functions may be carried out by the plant nucleolus. Further studies will be needed to elucidate the specific role of this subnuclear compartment in processes related to mammalian PML‐NBs and to unravel the role of SUMOylation in subnuclear protein targeting and/or function.

## Materials and methods

### Plant materials

*Nicotiana benthamiana* plants were grown on soil in a greenhouse with a 16 h light/8 h darkness photoperiod and temperatures of 25 and 20 °C, respectively.

### Plasmid constructs

In order to generate an eGFP destination vector for C‐terminally fused eGFP, the coding sequence of eGFP was amplified from pK7FWG2 56 (Oligonucleotide primers, eGFPfw: 5′‐GTCGACGGATCTGGTGTGAGCAAGGGCGAGGAGCTG‐3′; eGFPrev: 5′‐CTGCAGTCACTACTTGTACAGCTCGTCCATGC‐3′), subcloned into pCR^™^‐Blunt (Thermo Fisher Scientific, Waltham, MA, USA) and afterward excised using the restriction enzymes *Sal*I and *Pst*I. The fragment was eventually ligated into pRB35S 57. Coding sequences of human PML isoform VI 25 and of hPMLcs 8 were amplified via PCR using suitable oligonucleotide primers (PMLfw: 5′‐GAATTCAACAATGGAGCCTGCACCCGC‐3′; PMLrev: 5′‐GTCGACCCACAACGCGTTCCTCTC‐3′). After subcloning in pJET1.2/blunt using the CloneJET PCR Cloning Kit (Thermo Fisher Scientific), the respective fragments were excised using the restriction enzymes *Eco*RI and *Sal*I and inserted into the vector pRB35S‐eGFP.

The *S. tuberosum* COP1::RFP construct as well as the *A. thaliana* FIB2::RFP construct 27 was kindly provided by Prof. Michael Taliansky (The James Hutton Institute, Scotland UK).

### Transient expression in primary human fibroblast, *N*. *benthamiana*, and confocal laser scanning microscopy

Primary human foreskin fibroblasts (HFFs) with a small interfering RNA‐mediated knockdown of PML were cultured as described previously 58. For transient expression of PMLVI in HFFs, 3 × 10^5^ cells were seeded into six‐well dishes and transfected using the DNA transfection reagent FuGENE6 (Promega, Mannheim, Germany). FLAG‐tagged PML variants were detected by indirect immunofluorescence analysis using the monoclonal antibody anti‐FLAG 1804 (Sigma‐Aldrich, Deisenhofen, Germany) and Alexa Fluor 488‐conjugated secondary antibodies. DAPI staining was used to visualize the cell's nucleus. For transient expression approaches in plant cells, correspondingly transformed *Agrobacterium tumefaciens* (Strain C58C1) were pressure infiltrated as described 59, 60. Staining with DAPI and imaging of leaf samples using a Leica TCS SP5 II confocal laser scanning microscope (Leica Microsystems, Wetzlar, Germany) were conducted as described earlier 61. Statistical analysis of colocalization was carried out using Pearson's correlation coefficient as calculated by the las af software (Leica Microsystems) for Z‐stack micrographs covering the whole nucleus.

### Coimmunoprecipitation assays

In order to show SUMOylation of hPML::eGFP in plant cells, two *N. benthamiana* leaf disks (diameter appr. 2.5 cm) expressing hPML::eGFP, hPMLcs::eGFP, hPML3 m::eGFP, or free GFP were harvested 2 days after *Agrobacterium* infiltration. Samples were homogenized in 600‐μL of ice cold extraction buffer (100 mm Tris HCl pH 7.5, 300 mm NaCl, 10 mm DTT, 0.5 mm EDTA, 0.4% Triton X‐100, cOmplete ULTRA Tablets (Roche diagnostics, Mannheim, Germany)) using silicon carbide (Sigma‐Aldrich) as an abrasive. After incubating 20 min on ice, samples were centrifuged for 20 min at 15 900 ***g*** and 4 °C. To 400 μL of supernatant, 400 μL of extraction buffer devoid of Triton X‐100 but containing 10 μL GFP‐Trap^®^ magnetic bead slurry (Chromotek, Planegg‐Martinsried, Germany) was added. After 2 h of constant mixing at 4 °C, magnetic beads were washed twice in extraction buffer containing 0.01% Triton X‐100. Bound proteins were eluted by adding 70 μL Laemmli buffer and boiling for 10 min at 95 °C 62.

For proteomic analysis, the same amount of plant material as stated earlier was harvested from plants expressing PML::eGFP or free GFP, and four biological replicates of each sample were freshly homogenized in 1 mL of ice‐cold extraction buffer containing 100 mm Tris HCl pH 7.5, 300 mm NaCl, 10 mm DTT, 0.5 mm EDTA, 0.5% (v/v) NP‐40, cOmplete ULTRA Tablets (Roche diagnostics). After 20 min of incubation on ice, extracts were centrifuged for 10 min at 15 900 ***g*** and 4 °C. Supernatant was transferred into a new microcentrifugation tube and diluted with 1 mL of ice‐cold dilution buffer (100 mm Tris HCl pH 7.5, 300 mm NaCl, 10 mm DTT, 0.5 mm EDTA, cOmplete ULTRA Tablets (Roche diagnostics)) supplemented with 25 μL GFP‐Trap^®^ magnetic bead slurry (Chromotek). Samples were kept at 4 °C under constant mixing for 2 h, before beads were washed three times with cold dilution buffer with additional 0.1% NP‐40 and once with dilution buffer devoid of detergent. Immunoprecipitated proteins were eluted in 100 μL of 200 mm glycine pH 2.5. After extraction of beads, supernatant containing proteins was transferred into new microcentrifugation tubes and pH was neutralized using 20 μL Tris HCl pH 8.0.

### Protein polyacrylamide gel electrophoresis and western blot analysis

After extraction of magnetic beads, samples derived from immunoprecipitation were subjected to centrifugation for 3 min at 15 900 ***g***. Supernatant was loaded on a Bis/Tris Gel containing 12% acrylamide and subjected to electrophoresis. Proteins were then transferred onto a nitrocellulose membrane, which was afterward blocked for 1 h in blocking buffer (20 mm Tris HCl, 500 mm NaCl, 0.1% (v/v) Tween 20, 5% (w/v) milk powder). Detection was achieved by the use of suitable primary antibodies (Polyclonal anti‐AtSUMO‐1 antibody: Abcam, Cambridge, UK; polyclonal anti‐GFP antibody: Roche diagnostics) and secondary antibodies conjugated to horseradish peroxidase (Thermo Fisher Scientific) followed by enhanced chemiluminescence detection.

### Tryptic digest

Tryptic digest of previously extracted proteins was carried out based on the modified filter‐aided sample preparation (FASP) method 63. In short, eluted proteins were collected on 10 kDa Vivacon 500 Hydrosart^®^ membrane filters (Sartorius Stedim Biotech, Göttingen, Germany) by centrifugation at 15 900 ***g*** for 8 min, flow‐through was discarded. Reduction was performed by the addition of 250 μL of buffer containing 8 m urea, 100 mm triethylammonium bicarbonate buffer (TEAB), 20 mm DTT, and incubation for 30 min at room temperature under constant mixing. After centrifugation and discarding of flow‐through, alkylation of reduced proteins was achieved by addition of 250 μL of 8 m urea, 100 mm TEAB, 40 mm chloroacetamide, and incubation for 30 min at room temperature in the dark under constant mixing. After centrifugation, a washing step with 8 m urea and 100 mm TEAB was performed, before filters were transferred to new microcentrifugation tubes. For tryptic digest, 250 μL of 1 m urea, 50 mm TEAB, and 1 μg trypsin was added and samples were incubated overnight at 37 °C. Resulting peptides were retrieved by centrifugation and desalted on C18 stage tips. Prior to LC‐MS/MS analysis, peptides were concentrated on a centrifugal evaporator and resuspended in 0.1% trifluoroacetic acid (TFA).

### Nano‐LC‐MS/MS analysis

Peptides obtained by tryptic digestion were analyzed by an Orbitrap Fusion^™^ Tribrid^™^ mass spectrometer in connection with an UltiMate 3000 nano‐UHPLC system (Thermo Fisher Scientific). The samples were loaded and separated on PepMap100 columns (300 μm i.d. × 5 mm, 5 μm, 100 Å and 75 μm i.d. × 50 cm, 3 μm, 100 Å, respectively) with a 3–35% gradient of 0.1% formic acid in acetonitrile for 160 min at a flow rate of 300 nL·min^−1^ at 35 °C. Ionization of peptides was achieved using a nanospray Flex^™^ ion source (Thermo Fisher Scientific), precursor ions were analyzed at a scan range of 300–2000 *m*/*z*. The most intense peptides of each scan cycle were selected and fragmented by collision‐induced dissociation, resulting MS/MS scans were analyzed in the ion trap. Raw data files were evaluated using peaks7 (Bioinformatics Solutions Inc., Waterloo, ON, Canada) and a *N. benthamiana* proteome database (Sol Genomics Network, retrieved on 06.03.2015 64). The amino acid sequence of hPML::eGFP was additionally added to this database. Peptide identification was based on two trypsin miscleavages maximum and 10 ppm mass tolerance for survey scans. Carbamidomethylation was set as fixed modification, whereas oxidation of methionine residues was regarded optional. Resulting peptides were only considered as identified with a false discovery rate (FDR) below 1%. For label‐free quantification, two groups (free GFP control and PML::eGFP immunoprecipitation) of which each contained the respective four biological replicates were used. The samples were normalized by the total ion count (TIC) and only proteins with two or more unique peptides were included in the calculation. Finally, only proteins that were at least fivefold more abundant in the hPML::eGFP‐samples (with a significance of 13 ‐10lgP or higher) were included in the list used in this study. All mass spectrometry proteomics data have been deposited to the ProteomeXchange Consortium (http://proteomecentral.proteomexchange.org) via the PRIDE partner repository 65 with the dataset identifier PXD004254.

## Author contributions

US and TS conceived and designed the experiments; CEL performed all experiments in plant cells; MS and NR performed the analysis in mammalian cells; BA performed the MS‐analysis; CEL together with US and TS analyzed the data; CEL, TS, and US wrote the paper.

## Supporting information


**Table S1.** Complete list of proteins identified by hPML coimmunoprecipitation.
