# Supplementary material for: Human promyelocytic leukemia protein is targeted to distinct subnuclear domains in plant nuclei and colocalizes with nucleolar constituents in a SUMO‐dependent manner
Source: FEBS Open Bio. 2016 Oct 19;6(11):1141–54. doi: 10.1002/2211-5463.12134 (PMC5095151; doi:10.1002/2211-5463.12134)
Supplement: Supplementary file 1 — Table S1. Complete list of proteins identified by hPML coimmunoprecipitation. [file FEB4-6-1141-s001.pdf]

Accession	Significance (-10lgP)	Coverage (%)	#Peptides	#Unique	PTM	Sample Profile	Group Profile	Avg. Mass	Description
-	119.85	94	384	382	Y			89215	Homo sapiens|PML:GFP
Niben101Scf00573g05005.1	65.74	16	4	3	Y			38218	sp|A9WFT9|PDXS_CHLAA **** Pyridoxal biosynthesis lyase PdxS IPR001852 (Pyridoxine biosynthesis protein), IPR013785 (Aldolase-type TIM barrel) GO:0003824 (catalytic activity), GO:0008152 (metabolic process), GO:0042823 (pyridoxal ...
Niben101Scf04778g02012.1	53.33	2	2	2	N			98115	sp|Q6Z382|COPG2_ORYSJ ***- Coatomer subunit gamma-2 IPR009028 (Coatomer/calthrin adaptor appendage, C-terminal subdomain), IPR013041 (Coatomer/clathrin adaptor appendage, Ig-like subdomain), IPR016024 (Armadillo-type fold), IPR01...
Niben101Scf20652g01025.1	48.64	13	14	3	Y			127914	AT1G62020.1 ***- Coatomer, alpha subunit LENGTH=1216 IPR011990 (Tetratricopeptide-like helical domain), IPR016391 (Coatomer alpha subunit) GO:0005198 (structural molecule activity), GO:0005515 (protein binding), GO:0006886 (intra...
Niben101Scf01436g00001.1	45.15	10	2	2	N			30697	ref|XP_002509463.1| *-** structural constituent of cell wall, putative [Ricinus communis] gb|EEF50850.1| structural constituent of cell wall, putative [Ricinus communis] IPR003882 (Pistil-specific extensin-like protein), IPR00604...
Niben101Scf11995g00002.1	44.76	20	6	6	Y			39817	AT3G08030.1 ***- Protein of unknown function, DUF642 LENGTH=365 IPR006946 (Protein of unknown function DUF642), IPR008979 (Galactose-binding domain-like)
Niben101Scf01888g07004.1	44.52	39	11	11	Y			33189	sp|O74111|RFC3_BLAAD **** Replication factor C subunit 3 IPR003959 (ATPase, AAA-type, core), IPR008921 (DNA polymerase III, clamp loader complex, gamma/delta/delta subunit, C-terminal), IPR027417 (P-loop containing nucleoside tri...
Niben101Scf03949g06008.1	41.89	28	4	4	N			21052	gb|ACG38839.1| ***- snRK1-interacting protein 1 [Zea mays]
Niben101Scf14996g00009.1	39.55	36	14	12	Y			52712	sp|Q9M5L6|CATA_CAPAN **** Catalase IPR002226 (Catalase haem-binding site), IPR010582 (Catalase immune-responsive domain), IPR011614 (Catalase core domain), IPR018028 (Catalase, mono-functional, haem-containing), IPR020835 (Catala...
Niben101Scf00548g00009.1	38.55	4	3	3	N			96728	sp|Q9SSR4|PPR77_ARATH ***- Pentatricopeptide repeat-containing protein IPR002625 (Smr protein/MutS2 C-terminal), IPR002885 (Pentatricopeptide repeat), IPR011990 (Tetratricopeptide-like helical domain) GO:0005515 (protein binding)
Niben101Scf02280g05005.1	36.25	14	4	2	N			37617	sp|O04202|EIF3F_ARATH **** Eukaryotic translation initiation factor 3 subunit F IPR000555 (JAB1/MPN/MOV34 metalloenzyme domain), IPR024969 (Rpn11/EIF3F C-terminal domain), IPR027531 (Eukaryotic translation initiation factor 3 sub...
Niben101Scf03015g05008.1	35.89	26	10	3	Y			56788	AT5G15270.2 **** RNA-binding KH domain-containing protein LENGTH=548 IPR004087 (K Homology domain) GO:0003676 (nucleic acid binding), GO:0003723 (RNA binding)
Niben101Scf00271g06003.1	32.35	21	5	2	Y			31943	sp|Q9SA78|COPE1_ARATH ***- Coatomer subunit epsilon-1 IPR006822 (Coatomer, epsilon subunit) GO:0005198 (structural molecule activity), GO:0005515 (protein binding), GO:0006890 (retrograde vesicle-mediated transport, Golgi to ER)
Niben101Scf07691g00008.1	31.70	14	5	4	Y			57453	gb|KFB86097.1| ***- dihydrolipoamide dehydrogenase [Calothrix sp. 336/3] IPR013027 (FAD-dependent pyridine nucleotide-disulphide oxidoreductase), IPR016156 (FAD/NAD-linked reductase, dimerisation domain), IPR023753 (Pyridine nucl...
Niben101Scf01020g00014.1	30.84	17	5	5	N			35007	sp|Q93YF5|SUVH1_TOBAC **** Histone-lysine N-methyltransferase, H3 lysine-9 specific SUVH1 IPR003105 (SRA-YDG), IPR015947 (PUA-like domain) GO:0042393 (histone binding)
Niben101Scf23821g00012.1	30.48	6	2	2	N			36058	sp|B2VDG5|RLMI_ERWT9 --** Ribosomal RNA large subunit methyltransferase I IPR004398 (RNA methyltransferase, RsmD), IPR029063 (S-adenosyl-L-methionine-dependent methyltransferase) GO:0008168 (methyltransferase activity), GO:003116...
Niben101Scf05848g04014.1	29.97	8	3	3	N			56117	sp|A4QJA7|RPOB_AETCO **** DNA-directed RNA polymerase subunit beta IPR015712 (DNA-directed RNA polymerase, subunit 2) GO:0003677 (DNA binding), GO:0003899 (DNA-directed RNA polymerase activity), GO:0006351 (transcription, DNA-tem...
Niben101Scf00197g02006.1	29.32	20	20	2	Y			137138	AT1G62020.1 ***- Coatomer, alpha subunit LENGTH=1216 IPR011048 (Cytochrome cd1-nitrite reductase-like, haem d1 domain), IPR016391 (Coatomer alpha subunit) GO:0005198 (structural molecule activity), GO:0005515 (protein binding), G...
Niben101Scf07744g00017.1	29.29	13	3	2	N			19717	sp|B1ZA43|NUOD_METPB *-** NADH-quinone oxidoreductase subunit D IPR029014 (NiFe hydrogenase-like) GO:0016651 (oxidoreductase activity, acting on NAD(P)H), GO:0048038 (quinone binding), GO:0051287 (NAD binding), GO:0055114 (oxidat...
Niben101Scf05438g04003.1	29.28	6	2	2	Y			61304	sp|Q8RYM9|LAC2_ORYSJ ***- Laccase-2 IPR008972 (Cupredoxin)
Niben101Scf06267g03010.1	28.49	18	8	5	N			60200	AT2G17970.1 **** 2-oxoglutarate (2OG) and Fe(II)-dependent oxygenase superfamily protein LENGTH=507 IPR005123 (Oxoglutarate/iron-dependent dioxygenase), IPR027450 (Alpha-ketoglutarate-dependent dioxygenase AlkB-like) GO:0016491 (...
Niben101Scf10910g00006.1	28.04	19	7	7	Y			45149	AT1G26190.1 *-** Phosphoribulokinase / Uridine kinase family LENGTH=674 IPR006082 (Phosphoribulokinase), IPR027417 (P-loop containing nucleoside triphosphate hydrolase) GO:0005524 (ATP binding), GO:0005975 (carbohydrate metabolic...
Niben101Scf03576g01039.1	27.64	5	4	4	Y			110761	sp|P38935|SMBP2_HUMAN ***- DNA-binding protein SMUBP-2 IPR014001 (Helicase superfamily 1/2, ATP-binding domain), IPR027417 (P-loop containing nucleoside triphosphate hydrolase)
Niben101Scf11531g01010.1	27.11	7	3	2	N			41466	AT1G18870.1 *-*- isochorismate synthase 2 LENGTH=562
Niben101Scf02197g01014.1	25.04	30	16	3	Y			50352	AT1G77470.1 **** replication factor C subunit 3 LENGTH=369 IPR003959 (ATPase, AAA-type, core), IPR008921 (DNA polymerase III, clamp loader complex, gamma/delta/delta subunit, C-terminal), IPR027417 (P-loop containing nucleoside t...
Niben101Scf07382g01020.1	24.74	12	4	3	Y			49302	sp|Q02096|PGLR_PERAE **** Polygalacturonase IPR000743 (Glycoside hydrolase, family 28), IPR011050 (Pectin lyase fold/virulence factor) GO:0004650 (polygalacturonase activity), GO:0005975 (carbohydrate metabolic process)
Niben101Scf04181g00024.1	24.23	37	19	7	Y			67643	sp|Q67ZB3|FRL3_ARATH ***- FRIGIDA-like protein 3 IPR012474 (Frigida-like)

Niben101Scf03607g00009.1	24.15	9	4	4	N			57975	AT4G20130.1 ***- plastid transcriptionally active 14 LENGTH=483 IPR001214 (SET domain), IPR015353 (Rubisco LSMT, substrate-binding domain) GO:0005515 (protein binding)
Niben101Scf17442g00002.1	23.97	9	8	8	N			107768	sp|Q55738|GYRA_SYNY3 **** DNA gyrase subunit A IPR005743 (DNA gyrase, subunit A), IPR024946 (Arginine repressor C-terminal-like domain) GO:0003677 (DNA binding), GO:0003916 (DNA topoisomerase activity), GO:0003918 (DNA topoisomer...
Niben101Scf01237g03007.1	22.83	36	10	5	Y			29340	gb|ABD66598.1| **** iron-binding protein [Pyrus pyrifolia] IPR001519 (Ferritin), IPR008331 (Ferritin/DPS protein domain), IPR009078 (Ferritin-like superfamily) GO:0006826 (iron ion transport), GO:0006879 (cellular iron ion homeos...
Niben101Scf09321g00009.1	22.00	11	5	5	N			57579	sp|Q39247|2ABB_ARATH **** Serine/threonine protein phosphatase 2A 55 kDa regulatory subunit B beta isoform IPR000009 (Protein phosphatase 2A, regulatory subunit PR55), IPR015943 (WD40/YVTN repeat-like-containing domain) GO:000015...
Niben101Scf01281g02006.1	21.60	15	6	6	N			51633	sp|Q47N43|RUVB_THEFY --** Holliday junction ATP-dependent DNA helicase RuvB IPR010339 (TIP49, C-terminal), IPR027238 (RuvB-like), IPR027417 (P-loop containing nucleoside triphosphate hydrolase) GO:0003678 (DNA helicase activity),...
Niben101Scf04198g01001.1	21.51	9	8	8	Y			134908	sp|P51375|GLTB_PORPU **** Ferredoxin-dependent glutamate synthase IPR013785 (Aldolase-type TIM barrel), IPR029055 (Nucleophile aminohydrolases, N-terminal) GO:0003824 (catalytic activity), GO:0006537 (glutamate biosynthetic proce...
Niben101Scf01157g00004.1	21.21	34	18	2	Y			76024	sp|A6TWP7|FTSH2_ALKMQ **** ATP-dependent zinc metalloprotease FtsH 2 IPR003959 (ATPase, AAA-type, core), IPR005936 (Peptidase, FtsH), IPR027417 (P-loop containing nucleoside triphosphate hydrolase) GO:0004222 (metalloendopeptidas...
Niben101Scf15360g00006.1	21.14	32	11	4	Y			49986	dbj|BAF00138.1| **** RNA-binding protein like [Arabidopsis thaliana] IPR002344 (Lupus La protein), IPR011991 (Winged helix-turn-helix DNA-binding domain), IPR012677 (Nucleotide-binding alpha-beta plait domain) GO:0000166 (nucleot...
Niben101Scf00212g02005.1	20.35	8	5	5	N			69674	AT3G14900.1 ***- unknown protein; FUNCTIONS IN: molecular_function unknown; INVOLVED IN: embryo development; LOCATED IN: chloroplast; EXPRESSED IN: 19 plant structures; EXPRESSED DURING: 13 growth stages; Has 17135 Blast hits to ...
Niben101Scf01382g04008.1	20.03	34	28	5	Y			103539	sp|P40339|RFC4_YEAST *-** Replication factor C subunit 4 IPR012178 (DNA replication factor C, large subunit), IPR027417 (P-loop containing nucleoside triphosphate hydrolase) GO:0003677 (DNA binding), GO:0003689 (DNA clamp loader ...
Niben101Scf00414g09001.1	19.88	10	3	3	N			44541	sp|Q2NFZ6|RPOD_METST **** DNA-directed RNA polymerase subunit D IPR009025 (DNA-directed RNA polymerase, RBP11-like dimerisation domain) GO:0003677 (DNA binding), GO:0003899 (DNA-directed RNA polymerase activity), GO:0006351 (tran...
Niben101Scf05387g09003.1	19.67	15	3	3	N			23559	sp|Q5AAW3|DHH1_CANAL *-*- ATP-dependent RNA helicase DHH1 IPR001650 (Helicase, C-terminal), IPR027417 (P-loop containing nucleoside triphosphate hydrolase)
Niben101Scf01985g02007.1	19.18	8	4	4	N			61148	sp|Q4L530|HTRAL_STAHJ *-** Serine protease HtrA-like IPR001940 (Peptidase S1C), IPR009003 (Trypsin-like cysteine/serine peptidase domain) GO:0003824 (catalytic activity), GO:0004252 (serine-type endopeptidase activity), GO:000551...
Niben101Scf02354g12007.1	18.83	23	11	2	Y			60550	sp|A7SDW5|EIF3L_NEMVE **** Eukaryotic translation initiation factor 3 subunit L IPR019382 (Translation initiation factor 3 complex subunit L) GO:0003743 (translation initiation factor activity), GO:0005737 (cytoplasm), GO:0005852...
Niben101Scf01611g04026.1	18.75	7	4	4	N			79964	sp|Q8RXH2|NUP85_ARATH ***- Nuclear pore complex protein NUP85 IPR011502 (Nucleoporin Nup85-like)
Niben101Scf13569g00001.1	18.55	20	4	4	Y			36014	ref|WP_009711804.1| **** dihydroxynaphthoic acid synthetase [Desmospora sp. 8437] gb|EGK07457.1| naphthoate synthase [Desmospora sp. 8437] IPR001753 (Crotonase superfamily), IPR014748 (Crontonase, C-terminal), IPR029045 (ClpP/cro...
Niben101Scf03572g02005.1	18.38	9	4	4	N			53798	sp|Q8SS33|TCPQ_ENCCU ***- T-complex protein 1 subunit theta IPR002423 (Chaperonin Cpn60/TCP-1), IPR027409 (GroEL-like apical domain), IPR027410 (TCP-1-like chaperonin intermediate domain), IPR027413 (GroEL-like equatorial domain)...
Niben101Scf00539g02004.1	18.32	10	2	2	N			54839	gb|AES59797.2| *-*- LA-related protein 6 LA RNA-binding domain protein [Medicago truncatula] IPR011991 (Winged helix-turn-helix DNA-binding domain)
Niben101Scf12382g00017.1	18.13	22	9	2	Y			44831	sp|Q03164|KMT2A_HUMAN *-** Histone-lysine N-methyltransferase 2A IPR001214 (SET domain), IPR003105 (SRA-YDG), IPR003616 (Post-SET domain), IPR007728 (Pre-SET domain), IPR015947 (PUA-like domain), IPR025794 (Histone H3-K9 methyltr...
Niben101Scf01086g00013.1	17.52	13	7	7	N			77275	AT4G11610.1 **** C2 calcium/lipid-binding plant phosphoribosyltransferase family protein LENGTH=1011 IPR000008 (C2 domain) GO:0005515 (protein binding)
Niben101Scf02182g05003.1	17.50	3	2	2	N			96451	AT1G31230.1 **** aspartate kinase-homoserine dehydrogenase i LENGTH=911 IPR011147 (Bifunctional aspartokinase/homoserine dehydrogenase I), IPR016040 (NAD(P)-binding domain) GO:0004072 (aspartate kinase activity), GO:0004412 (homo...
Niben101Scf00565g00013.1	17.49	36	12	2	Y			39400	AT1G01300.1 ***- Eukaryotic aspartyl protease family protein LENGTH=485 IPR001461 (Aspartic peptidase), IPR021109 (Aspartic peptidase domain) GO:0004190 (aspartic-type endopeptidase activity), GO:0006508 (proteolysis)
Niben101Scf07109g05004.1	17.26	24	3	3	N			17476	AT4G35060.1 **** Heavy metal transport/detoxification superfamily protein LENGTH=153 IPR006121 (Heavy metal-associated domain, HMA) GO:0030001 (metal ion transport), GO:0046872 (metal ion binding)
Niben101Scf00013g03001.1	16.99	3	2	2	N			100448	sp|B9L3S8|FTSH2_THERP *-** ATP-dependent zinc metalloprotease FtsH 2 IPR000642 (Peptidase M41), IPR003959 (ATPase, AAA-type, core), IPR027417 (P-loop containing nucleoside triphosphate hydrolase) GO:0004222 (metalloendopeptidase ...
Niben101Scf06884g00001.1	16.81	9	6	5	N			74623	sp|Q5GUR8|RHLB_XANOR *-** ATP-dependent RNA helicase RhlB IPR001650 (Helicase, C-terminal), IPR012562 (GUCT), IPR014001 (Helicase superfamily 1/2, ATP-binding domain), IPR014014 (RNA helicase, DEAD-box type, Q motif), IPR027417 (...
Niben101Scf03390g08002.1	16.79	37	16	2	Y			54148	sp|O22478|IMA_SOLLC ***- Importin subunit alpha IPR002652 (Importin-alpha, importin-beta-binding domain), IPR016024 (Armadillo-type fold), IPR024931 (Importin subunit alpha) GO:0005488 (binding), GO:0005515 (protein binding), GO:...

Niben101Scf07391g04027.1	16.76	22	9	2	Y			53623	sp|Q6YZ54|RFC3_ORYSJ **** Replication factor C subunit 3 IPR003959 (ATPase, AAA-type, core), IPR008921 (DNA polymerase III, clamp loader complex, gamma/delta/delta subunit, C-terminal), IPR027417 (P-loop containing nucleoside tri...
Niben101Scf03538g02003.1	16.07	9	5	5	N			78572	sp|P43672|UUP_ECOLI **** ABC transporter ATP-binding protein uup IPR003439 (ABC transporter-like), IPR027417 (P-loop containing nucleoside triphosphate hydrolase) GO:0005524 (ATP binding), GO:0016887 (ATPase activity)
Niben101Scf08523g00013.1	15.85	10	4	4	N			55278	AT3G27190.1 **** uridine kinase-like 2 LENGTH=483 IPR000764 (Uridine kinase like), IPR027417 (P-loop containing nucleoside triphosphate hydrolase), IPR029057 (Phosphoribosyltransferase-like) GO:0004849 (uridine kinase activity), ...
Niben101Scf06172g01003.1	15.85	8	4	3	N			69527	sp|Q8L4D8|IQD31_ARATH ***- Protein IQ-DOMAIN 31 IPR000048 (IQ motif, EF-hand binding site), IPR025064 (Domain of unknown function DUF4005) GO:0005515 (protein binding)
Niben101Scf15549g00002.1	15.85	9	3	3	N			46712	sp|Q8KCJ0|HEM2_CHLTE ***- Delta-aminolevulinic acid dehydratase IPR001731 (Porphobilinogen synthase), IPR013785 (Aldolase-type TIM barrel) GO:0003824 (catalytic activity), GO:0004655 (porphobilinogen synthase activity), GO:003301...
Niben101Scf08698g02020.1	15.82	14	5	5	N			61028	AT2G34640.1 ***- plastid transcriptionally active 12 LENGTH=527
Niben101Scf04267g00016.1	15.67	17	9	2	Y			37373	sp|Q68WZ0|RUVB_RICTY -*** Holliday junction ATP-dependent DNA helicase RuvB IPR008921 (DNA polymerase III, clamp loader complex, gamma/delta/delta subunit, C-terminal), IPR027417 (P-loop containing nucleoside triphosphate hydrola...
Niben101Scf01181g00019.1	15.50	36	7	4	Y			33681	sp|Q8SRB0|GBLP_ENCCU **** Guanine nucleotide-binding protein subunit beta-like protein IPR015943 (WD40/YVTN repeat-like-containing domain), IPR020472 (G-protein beta WD-40 repeat) GO:0005515 (protein binding)
Niben101Scf07008g03009.1	15.37	10	6	2	N			83660	AT4G12770.1 *-*- Chaperone DnaJ-domain superfamily protein LENGTH=891 IPR001623 (DnaJ domain)
Niben101Scf05056g02015.1	14.43	46	11	4	Y			28943	sp|P40937|RFC5_HUMAN **** Replication factor C subunit 5 IPR003959 (ATPase, AAA-type, core), IPR008921 (DNA polymerase III, clamp loader complex, gamma/delta/delta subunit, C-terminal), IPR027417 (P-loop containing nucleoside tri...
Niben101Scf06218g00003.1	14.30	39	12	4	Y			43433	sp|Q2HJ81|TBB6_BOVIN ***- Tubulin beta-6 chain IPR000217 (Tubulin), IPR023123 (Tubulin, C-terminal) GO:0003924 (GTPase activity), GO:0005200 (structural constituent of cytoskeleton), GO:0005525 (GTP binding), GO:0005874 (microtub...
Niben101Scf07839g00002.1	14,09,2016	3	2	2	N			67570	sp|Q9SIS3|MA656_ARATH **** 65-kDa microtubule-associated protein 6 IPR007145 (Microtubule-associated protein, MAP65/Ase1/PRC1) GO:0000226 (microtubule cytoskeleton organization), GO:0000910 (cytokinesis), GO:0008017 (microtubule ...
Niben101Scf05078g04001.1	13.91	3	3	2	N			159819	AT1G24300.1 *-*- GYF domain-containing protein LENGTH=1495 IPR003169 (GYF) GO:0005515 (protein binding)
Niben101Scf04623g02007.1	13.86	14	2	2	N			21608	sp|Q0UBQ5|NOP16_PHANO --*- Nucleolar protein 16 IPR019002 (Ribosome biogenesis protein Nop16)
Niben101Scf11084g02025.1	13.82	8	3	3	N			61524	AT2G41500.1 ***- WD-40 repeat family protein / small nuclear ribonucleoprotein Prp4p-related LENGTH=554 IPR014906 (Pre-mRNA processing factor 4 (PRP4)-like), IPR015943 (WD40/YVTN repeat-like-containing domain), IPR020472 (G-prote...
Niben101Scf02908g01016.1	13.69	19	4	2	N			31241	ref|WP_026735795.1| ***- molecular chaperone DnaJ [Fischerella sp. PCC 9605] IPR021788 (Protein of unknown function DUF3353)
Niben101Scf07019g01004.1	13.57	8	3	3	N			65524	AT2G17890.1 **** calcium-dependent protein kinase 16 LENGTH=571 IPR011009 (Protein kinase-like domain), IPR011992 (EF-hand domain pair) GO:0004672 (protein kinase activity), GO:0004674 (protein serine/threonine kinase activity), ...
Niben101Scf07629g00001.1	13.47	14	3	2	N			29001	sp|Q9SIU2|NCBP1_ARATH *-** Nuclear cap-binding protein subunit 1 IPR016024 (Armadillo-type fold) GO:0005488 (binding), GO:0016070 (RNA metabolic process)
Niben101Scf06105g03006.1	13.46	12	4	4	N			40398	sp|Q9C652|RBP1_ARATH --** RNA-binding protein 1 IPR012677 (Nucleotide-binding alpha-beta plait domain) GO:0000166 (nucleotide binding), GO:0003676 (nucleic acid binding)
Niben101Scf01326g08021.1	13.09	11	6	5	N			67092	sp|Q8L4D8|IQD31_ARATH ***- Protein IQ-DOMAIN 31 IPR000048 (IQ motif, EF-hand binding site), IPR025064 (Domain of unknown function DUF4005) GO:0005515 (protein binding)

Table S1: Complete list of proteins identified by hPML coimmunoprecipitation. All listed proteins were identified as significantly more abundant in one sample group by label-free quantification. **Accession** number is adopted from the *Nicotiana benthamiana* proteome database, **Significance (-10lgP)** describes the calculated significance of the identification, **Coverage (%)** indicates the percentage of mass spectrometrically covered amino acid sequence of the respective protein, **#Peptides** shows how many peptides could be identified, **#Unique** shows how many of the identified peptides could be uniquely assigned to the respective protein, **PTM** indicates the presence (Y) or absence (N) of identified posttranslational modifications, **Sample and Group Profile** indicate the abundance (red - high, green - low) of the protein in the respective sample/group, **Avg. Mass** shows the calculated mass of the protein in Dalton, the **Description** was adopted from the *Nicotiana benthamiana* proteome database. Complete proteomics data have been deposited to the ProteomeXchange Consortium with the identifier PXD004254.
